# COVID-19-related outcomes in immunocompromised patients: A nationwide study in Korea

**DOI:** 10.1371/journal.pone.0257641

**Published:** 2021-10-01

**Authors:** Moon Seong Baek, Min-Taek Lee, Won-Young Kim, Jae Chol Choi, Sun-Young Jung

**Affiliations:** 1 Department of Internal Medicine, Chung-Ang University Hospital, Chung-Ang University College of Medicine, Seoul, Republic of Korea; 2 College of Pharmacy, Chung-Ang University, Seoul, Republic of Korea; 3 Department of Global Innovative Drugs, Graduate School of Chung-Ang University, Seoul, Republic of Korea; University of Toledo, UNITED STATES

## Abstract

**Background:**

Given the rapid increased in confirmed coronavirus disease 2019 (COVID-19) and related mortality, it is important to identify vulnerable patients. Immunocompromised status is considered a risk factor for developing severe COVID-19. We aimed to determine whether immunocompromised patients with COVID-19 have an increased risk of mortality.

**Method:**

The groups’ baseline characteristics were balanced using a propensity score-based inverse probability of treatment weighting approach. Odds ratios (ORs) and their 95% confidence intervals (CIs) were calculated for the risks of in-hospital mortality and other outcomes according to immunocompromised status using a multivariable logistic regression model. We identified immunocompromised status based on a diagnosis of malignancy or HIV/AIDS, having undergone organ transplantation within 3 years, prescriptions for corticosteroids or oral immunosuppressants for ≥30 days, and at least one prescription for non-oral immunosuppressants during the last year.

**Results:**

The 6,435 COVID-19 patients (≥18 years) included 871 immunocompromised (13.5%) and 5,564 non-immunocompromised (86.5%). Immunocompromised COVID-19 patients were older (60.1±16.4 years vs. 47.1±18.7 years, absolute standardized mean difference: 0.738). The immunocompromised group had more comorbidities, a higher Charlson comorbidity index, and a higher in-hospital mortality rate (9.6% vs. 2.3%; p < .001). The immunocompromised group still had a significantly higher in-hospital mortality rate after inverse probability of treatment weighting (6.4% vs. 2.0%, p < .001). Multivariable analysis adjusted for baseline imbalances revealed that immunocompromised status was independently associated with a higher risk of mortality among COVID-19 patients (adjusted odds ratio [aOR]: 2.09, 95% CI: 1.62–2.68, p < .001).

**Conclusions:**

Immunocompromised status among COVID-19 patients was associated with a significantly increased risk of mortality.

## Introduction

The coronavirus disease 2019 (COVID-19) pandemic is caused by the severe acute respiratory syndrome coronavirus 2 (SARS-CoV-2) and has emerged as a global threat to healthcare systems. The World Health Organization (WHO) estimates that the pandemic has involved >30 million COVID-19 cases and nearly 1 million deaths worldwide [1]. Given the rapid increases in confirmed COVID-19 cases and related mortality, it is important to identify vulnerable patients. Previous studies have indicated that the risk of severe COVID-19 is related to older age and underlying diseases, such as hypertension or diabetes [2–7].

Viral infections in immunocompromised patients are more likely to progress to severe disease [8], and immunocompromised status linked to malignancy or transplantation is considered a risk factor for developing severe COVID-19 [9–11]. However, conflicting results have been reported, as Gisondi et al. reported similar outcomes among COVID-19 patients regardless of whether they received immunosuppressive treatment [12]. In addition, Holcomb et al. reported that risks of COVID-19 and poor outcomes were rarely affected by immunomodulatory treatment [13]. A meta-analysis of 3,027 patients with SARS-CoV-2 infection revealed that poor COVID-19-related outcomes were associated with age of >65 years and comorbidities (including hypertension and diabetes), but not malignancy [14]. In this setting, immunosuppressed status can be related to acquired immunodeficiency syndrome (AIDS), hematological or solid malignancies, organ transplantation, and immunosuppressive medications [15]. Furthermore, immunocompromised patients are older and have more underlying diseases than non-immunocompromised hosts patients, although there is insufficient evidence regarding whether immunocompromised status is associated with more severe SARS-CoV-2 infection and/or mortality [16]. Therefore, the present study aimed to investigate whether pre-existing immunocompromised status was associated with poorer outcomes among Korean patients with SARS-CoV-2 infection.

## Materials and methods

### Data source

This retrospective population-based cohort study evaluated COVID-19 cases from the Health Insurance Review and Assessment Service (HIRA) database. In South Korea, the National Health Insurance system is a single-payer and compulsory healthcare insurance provider, and medical expenses related to COVID-19 are reimbursed by the HIRA. Claims data were released as part of the #OpenData4Covid19 project, which is a global COVID-19 research collaboration that is jointly conducted by the Ministry of Health and Welfare of Korea and HIRA [17]. This study’s protocol was approved by the Chung-Ang University Bioethics Committee (1041078-202005-HR-126-01), which waived the requirement for informed consent.

The first case of COVID-19 in South Korea was confirmed on January 20, 2020, and this study analyzed claims data from COVID-19 patients that were entered into the HIRA by May 15, 2020. The HIRA dataset is composed of six domains (general information, healthcare services, diagnosis, outpatient prescriptions, drug information, and provider information) [18]. Diagnostic codes were from the Korean Classification of Disease, 7th edition (KCD-7), which is a modification of the International Classification of Disease, 10th revision (ICD-10). Prescribed drugs were identified using Anatomical Therapeutic Chemical codes and HIRA general name codes.

### Study design and definitions

During the study period, 234,427 subjects underwent testing for COVID-19 using a reverse transcription polymerase chain reaction assay and an upper respiratory tract specimen [19]. Cases of COVID-19 were identified using the KCD-7 disease codes (B34.2, B97.2, U18, U18.1, and U07.1). The present study included patients with COVID-19 who were ≥18 years old and only patients identified as confirmed COVID-19 cases based on positive nasopharyngeal swabs tested using real-time reverse transcription-polymerase chain reaction assays [20]. We identified immunocompromised status based on a diagnosis of malignancy or HIV/AIDS, having undergone organ transplantation within 3 years, prescriptions for corticosteroids or oral immunosuppressants for ≥30 days during the last year, and at least one prescription for non-oral immunosuppressants during the last year (S1 Table). Immunosuppressant drugs included chemotherapy agents, biologic drugs, and immunomodulators, with detailed information shown in S1 Table. In order to identify for a severe population, we defined subjects who qualify for two or more immunocompromised status as ≥2 causes.

Claims were used to retrieve information regarding age, sex, Charlson comorbidity index (CCI), comorbidities, and region of residence. Comorbidities were measured using the CCI and identified using diagnostic codes from <1 year before the COVID-19 testing. Hypertension was identified based on the prescription of antihypertensive drugs within the last year. The Republic of Korea consists of 17 administrative districts that are classified into five regions: Region A (Seoul, Gyeonggi-do, and Incheon), Region B (Daejeon, Sejong, Chungcheongbuk-do, Chungcheongnam-do, and Gangwon-do), Region C (Daegu and Gyeongsangbuk-do), Region D (Busan, Ulsan, and Gyeongsangnamdo), and Region E (Jeollabuk-do, Gwangju, Jeollanam-do, and Jeju Special Self-Governing Province). Procedures related to COVID-19 included conventional oxygen therapy (M0040), high flow nasal cannula (M0046), mechanical ventilation (M5850, M5857, M5858, and M5860), extracorporeal membrane oxygenation (O1901–O1904), renal replacement therapy (O7051–7054), cardiopulmonary resuscitation (M5873–M5877). As an additional COVID-19 related outcome, we identified acute heart failure using KCD7 disease codes (I110, I130, I132, I255, I420, I425, I428, I429, I43, I50). Prescribed vasopressors included norepinephrine, vasopressin, dopamine, and dobutamine.

### Outcomes

The primary outcome was in-hospital mortality. The secondary outcomes were the occurrence of acute heart failure and the use of oxygen therapy, high flow nasal cannula, mechanical ventilation, ECMO, vasopressors, or renal replacement therapy.

### Statistical analysis

Continuous variables were presented as mean±standard deviations and categorical variables were presented as number (%). The characteristics of the immunocompromised and non-immunocompromised groups were compared using the absolute standardized mean difference (aSMD), with adequate balance defined as an aSMD of <0.1. Given that significant differences were observed in the baseline characteristics, we performed inverse probability of treatment weighting (IPTW) using propensity scores (PSs) for immunocompromised status that were calculated using a multivariable logistic regression model. The IPTW method creates a pseudo-population where baseline covariates are balanced using a PS function that predicts the probability of exposure assignments conditional on measured covariates. Owing to the relatively small number of immunocompromised patients in our study population, we chose to use the IPTW method. The method has the advantage of keeping larger observations in the analysis compared with PS matching and minimizing systematic differences between exposed and unexposed subjects compared with using PS adjustment or stratification. However, there could be an issue of variance inflation by extreme weights as a direct result of PS; in this study, we trimmed 10% from each tail of the PS distribution to improve the IPTW precision and to reduce bias related to unmeasured confounders [21]. The adjusted odds ratios (aORs) and 95% confidence intervals (CIs) for in-hospital mortality and other secondary outcomes were calculated according to immunocompromised status using multivariable logistic regression models, which were adjusted for age, region of residence, and comorbidities. The time from hospital admission to death was calculated according to immunocompromised status using IPTW-adjusted Kaplan-Meier curves and the log-rank test. Differences were considered statistically significant at p-values of <0.05 and all analyses were performed using SAS Enterprise Guide software (version 7.1).

## Results

The 6,435 adult patients with COVID-19 included 871 immunocompromised patients (13.5%) and 5,564 non-immunocompromised patients (86.5%) (S1 Fig). Immunocompromised COVID-19 patients were older (mean age: 60.1±16.4 years vs. 47.1±18.7 years) and both groups had high proportions of female patients (62.2% vs. 58.2%). The immunocompromised group had more comorbidities and a higher CCI. Most COVID-19 cases were in Region C (Daegu and Gyeongsangbuk-do) (Table 1).

**Table 1:** Baseline characteristics of COVID-19 patients.

	COVID-19 patients before weighting			COVID-19 patients after IPTW		
Variable	Immunocompromised	Non- immunocompromised	aSMD	Immunocompromised	Non- immunocompromised	aSMD
	(n = 871)	(n = 5,564)		(n = 5,186)	(n = 5,188)	
**Age**	60.1±16.4	47.1±18.7	0.738	57.3±16.7	46.7 ±18.3	0.164
18–39 years	100 (11.5)	2,124 (38.2)		18.9%	36.5%	
40–59 years	303 (34.8)	1,924 (34.6)		50.0%	36.4%	
60–79 years	364 (41.8)	1,248 (22.4)		23.9%	22.4%	
≥80 years	104 (11.9)	268 (4.8)		7.2%	4.7%	
**Sex**			0.082			0.065
Male	329 (37.8)	2,325 (41.8)		35.7%	38.9%	
Female	542 (62.2)	3,239 (58.2)		64.3%	61.2%	
**Comorbidities**						
Diabetes	294 (33.8)	936 (16.8)	0.397	20.4%	15.7%	0.122
Hypertension	377 (43.3)	1,305 (23.5)	0.430	27.2%	23.2%	0.094
Myocardial infarction	33 (3.8)	69 (1.2)	0.163	1.2%	0.9%	0.026
Congestive heart failure	95 (10.9)	207 (3.7)	0.279	4.3%	2.7%	0.086
Cerebrovascular disease	123 (14.1)	397 (7.1)	0.228	7.0%	6.7%	0.010
Chronic pulmonary disease	371 (42.6)	1,414 (25.4)	0.369	27.8%	25.9%	0.042
Chronic liver disease	368 (42.3)	1,072 (19.3)	0.514	21.5%	17.7%	0.097
Chronic renal disease	37 (4.3)	96 (1.7)	0.149	2.2%	1.3%	0.066
**Charlson comorbidity index**	3.6±2.8	1.2±1.6	1.084	2.9±2.4	1.0±1.3	0.294
0–2	358 (41.1)	4,677 (84.1)		62.5%	87.5%	
3–5	339 (38.9)	746 (13.4)		28.9%	11.6%	
≥6	174 (20.0)	141 (2.5)		8.6%	0.9%	
Region*			0.215			0.153
A	127 (14.6)	1,050 (18.9)		16.5%	15.6%	
B	59 (6.8)	486 (8.7)		8.6%	8.5%	
C	596 (68.4)	3,411 (61.3)		63.9%	64.4%	
D	46 (5.3)	456 (8.2)		6.5%	8.6%	
E	43 (4.9)	161 (2.9)		4.6%	2.9%	

Data are shown as number (%) or mean±standard deviation. COVID-19: Coronavirus disease 2019; IPTW: Inverse probability of treatment weighting; aSMD: Absolute standardized mean difference.

*The Republic of Korea consists of 17 administrative districts that are classified into give regions: Region A (Seoul, Gyeonggi-do, and Incheon), Region B (Daejeon, Sejong, Chungcheongbuk-do, Chungcheongnam-do, and Gangwon-do), Region C (Daegu and Gyeongsangbuk-do), Region D (Busan, Ulsan, and Gyeongsangnamdo), and Region E (Jeollabuk-do, Gwangju, Jeollanam-do, and Jeju Special Self-Governing Province).

**Fig 1:**
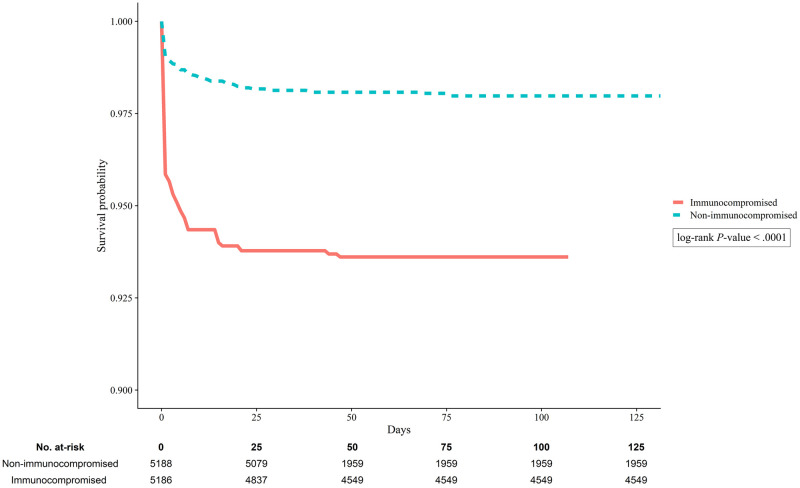
Kaplan-Meier survival curves for patients with COVID-19 according to immunocom-promised status after the inverse probability of treatment weighting (log-rank P-value <0.001).

The COVID-19-related outcomes according to immunocompromised status are shown in Table 2. The in-hospital mortality rates were 9.6% among immunocompromised patients and 2.3% among non-immunocompromised patients (p < .001). After the PS-based IPTW, the immunocompromised group still had a significantly higher in-hospital mortality rate (6.4% vs. 2.0%; p < .001). Furthermore, the immunocompromised group had a significantly greater probability of in-hospital mortality (estimated overall survival probability: 0.936 vs. 0.980, p < .001) (Fig 1). The multivariable analysis, which was adjusted for baseline imbalances, also revealed that immunocompromised status was independently associated with a higher risk of mortality (aOR: 2.09, 95% CI: 1.62–2.68, p < .001) (Table 3). Furthermore, immunocompromised status was an independent risk factor for conventional oxygen therapy use, mechanical ventilation use, and acute heart failure.

**Table 2:** Outcomes of patients with COVID-19 according to immunocompromised status.

	COVID-19 patients before weighting			COVID-19 patients after IPTW		
Outcomes	Immunocompromised	Non- immunocompromised	P-value	Immunocompromised	Non- immunocompromised	P-value
	(n = 871)	(n = 5,564)		(n = 5,186)	(n = 5,188)	
**In-hospital mortality**	84 (9.6)	127 (2.3)	< .001	330 (6.4)	101 (2.0)	< .001
**Conventional oxygen therapy**	229 (26.3)	678 (12.2)	< .001	973 (18.8)	608 (11.7)	< .001
**High flow nasal cannula**	63 (7.2)	119 (2.1)	< .001	249 (4.8)	105 (2.0)	< .001
**Mechanical ventilation**	45 (5.2)	82 (1.5)	< .001	138 (2.7)	69 (1.3)	< .001
**ECMO**	7 (0.8)	14 (0.3)	0.017	22 (0.4)	10 (0.2)	0.034
**Vasopressor use**	56 (6.4)	98 (1.8)	< .001	179 (3.5)	91 (1.8)	< .001
**Renal replacement therapy**	12 (1.4)	18 (0.3)	0.003	28 (0.6)	11 (0.2)	0.007
**Acute heart failure**	83 (9.5)	299 (5.4)	< .001	469 (9.0)	277 (5.4)	< .001

Data are shown as number (%).

COVID-19: Coronavirus disease 2019; IPTW: Inverse probability of treatment weighting; ECMO: Extracorporeal membrane oxygenation.

**Table 3:** Risks of outcomes among immunocompromised patients after inverse probability of treatment weighting.

Outcomes	Unadjusted OR	*p*-value	Adjusted OR*	*p*-value
	(95% CI)		(95% CI)	
**In-hospital mortality**	3.40 (2.71–4.26)	< .001	2.09 (1.62–2.68)	< .001
**Conventional oxygen therapy**	1.74 (1.56–1.94)	< .001	1.19 (1.05–1.35)	0.007
**High flow nasal cannula**	2.44 (1.94–3.07)	< .001	1.28 (1.00–1.65)	0.055
**Mechanical ventilation**	2.04 (1.52–2.73)	< .001	1.06 (1.05–1.07)	0.018
**ECMO**	2.17 (1.04–4.51)	0.038	2.17 (1.02–4.63)	0.045
**Vasopressor use**	2.01 (1.56–2.59)	< .001	1.04 (0.79–1.38)	0.773
**Renal replacement therapy**	2.48 (1.25–4.91)	0.010	1.26 (0.61–2.63)	0.534
**Acute heart failure**	1.76 (1.51–2.05)	< .001	1.39 (1.18–1.64)	< .001

IPTW: Inverse probability of treatment weighting; OR: Odds ratio; CI: Confidence interval; ECMO: Extracorporeal membrane oxygenation.

* Adjusted for Charlson Comorbidity Index, age, and region.

S2 Table shows the outcomes according to the various causes of immunocompromised status. Furthermore, with the exceptions of HIV/AIDS and organ transplantation, the adjusted ORs for the various outcomes were calculated in most subgroups after PS-based IPTW (Table 4). As a cause of immunocompromised status, malignancy was not an independent risk factor for in-hospital mortality among COVID-19 patients (aOR: 1.24, 95% CI: 0.92–1.66, p = 0.153), and solid tumors (accounted for 503 of 515 malignancies) were also not an independent risk factor for in-hospital mortality (S3 Table). However, corticosteroid use was an independent risk factor for in-hospital mortality (aOR: 3.36, 95% CI: 2.48–4.54, p < .001) and patients with ≥2 causes of their immunocompromised status had a significantly increased risk of in-hospital mortality (aOR: 2.79, 95% CI: 1.80–4.34, p < .001).

**Table 4:** Risks of outcomes in the subgroups of immunocompromised COVID-19 patients after IPTW.

Outcomes	Malignancy (n = 3,343)		Use of corticosteroids (n = 1,754)		Use of immunosuppressants (n = 433)		≥2 causes (n = 444)	
	Adjusted OR*	*p*-value	Adjusted OR	*p*-value	Adjusted OR	*p*-value	Adjusted OR	*p*-value
	(95% CI)		(95% CI)		(95% CI)		(95% CI)	
**In-hospital mortality**	1.24	0.153	3.36	< .001	1.19	0.495	2.79	< .001
	(0.92–1.66)		(2.48–4.54)		(0.73–1.93)		(1.80–4.34)	
**Conventional oxygen therapy**	1.02	0.829	1.47	< .001	0.08	0.027	2.10	< .001
	(0.88–1.18)		(1.25–1.72)		(0.01–0.75)		(1.61–2.74)	
**High flow nasal cannula**	1.42	0.210	1.12	0.513	1.09	0.557	1.71	0.020
	(1.35–1.49)		(0.80–1.58)		(0.82–1.46)		(1.09–2.68)	
**Mechanical ventilation**	1.25	0.043	0.17	< .001	1.26	0.331	1.01	0.986
	(1.17–1.34)		(0.07–0.40)		(0.79–2.03)		(0.47–2.17)	
**ECMO**	NA		NA		0.78	0.504	NA	
					(0.37–1.64)			
**Vasopressor use**	1.23	0.179	1.21	0.294	NA	0.966	2.78	< .001
	(0.91–1.67)		(0.85–1.73)				(1.74–4.43)	
**Renal replacement therapy**	1.51	0.053	NA		0.70	0.220	1.85	0.017
	(1.33–1.72)				(0.40–1.23)		(1.50–2.27)	
**Acute heart failure**	1.45	< .001	1.28	0.029	0.34	0.003	1.30	0.182
	(1.21–1.75)		(1.03–1.59)		(0.17–0.69)		(0.88–1.92)	

IPTW: Inverse probability of treatment weighting; OR: Odds ratio; CI: Confidence interval; ECMO: Extracorporeal membrane oxygenation; NA: Not applicable.

* Adjusted for Charlson comorbidity index, age, and region.

## Discussion

This nationwide study using propensity score-based IPTW evaluated whether immunocompromised status was associated with adverse outcomes among South Korean patients with COVID-19. The results revealed that immunocompromised patients had a significantly higher rate of in-hospital mortality, even after the PS-based IPTW. Furthermore, immunocompromised patients had a 2.09-fold higher risk of in-hospital mortality (aOR: 2.09, 95% CI: 1.62–2.68), even after adjusting for age, comorbidities, and region. Moreover, immunocompromised patients had an increased likelihood of severe COVID-19, based on the rates of mechanical ventilation use, renal replacement therapy use, and acute heart failure.

Although our findings indicate that immunosuppression is a risk factor for severe COVID-19 and/or death, previous studies have revealed conflicting evidence regarding this relationship. A systematic review by Minotti et al. revealed that immunosuppression may be not associated with more severe COVID-19, as immunosuppression tended to be associated with more favorable outcomes, relative to other comorbidities [22]. In contrast, a systematic review and meta-analysis by Gao et al. suggested that immunosuppression was associated with a non-significantly increased risk of severe COVID-19 [23]. These conflicting findings might be related to different definitions of immunosuppression [16], and the fact that most studies only included a small number of immunocompromised patients with various underlying causes. However, our study included a relatively large sample of immunocompromised patients and the logistic regression model was adjusted for potential confounding factors, such as age and other comorbidities. Moreover, we included immunocompromised patients according to a definition that has been validated in large databases [15].

Fung et al. reported that malignancy and solid-organ transplantation might be risk factors for severe COVID-19 and death [24]. Razanamahery et al. also evaluated various causes of immunosuppression, but reported no significant differences [25]. Our findings suggest that corticosteroid users had an increased risk of severe COVID-19 and death (aOR: 3.36, 95% CI: 2.48–4.54), although malignancy or treatment using immunosuppressive agents were not associated with poor outcomes. Systemic corticosteroid treatments have potent anti-inflammatory and immunomodulatory properties and are widely used to treat bronchial asthma, inflammatory bowel disease, and rheumatoid arthritis [26]. However, corticosteroid use is associated with an increased risk of adverse events [27], especially infectious complications among patients who are receiving prednisone at >10 mg/day or a cumulative dose of >700 mg [28]. In addition, Li et al. reported that high-dose corticosteroid treatment (prednisone at >1 mg/kg/day) was associated with a high risk of death among patients with severe COVID-19 [3]. Thus, although dexamethasone is an effective drug for treating COVID-19 [29], long-term or high-dose corticosteroid treatment should be used cautiously to avoid poor outcomes in the COVID-19 era.

Although we found that malignancy, as a cause of immunosuppression, was not associated with an increased risk of death, this result should be interpreted cautiously. A large meta-analysis that included 37,807 patients with COVID-19 and 2,034 deaths revealed that cancer was associated with an increased risk of all-cause mortality (RR: 1.66, 95% CI: 1.33–2.07) [30]. Furthermore, some case-control studies revealed that patients with cancer appeared to have an increased risk of death or severe disease, even after adjustment for potential confounders [7, 9, 10]. However, various factors might influence this relationship, including cancer stage and metastasis, as Kuderer et al. reported that patients who experienced cancer progression had a 5.2-fold higher risk of death than patients who were in remission [31]. Unfortunately, we did not have access to detailed information regarding cancer stage or recent anti-cancer treatments. Nevertheless, we observed that the co-existence of ≥2 causes of immunosuppression was associated with a 2.79-fold higher risk of mortality (Table 4), and we speculate that the risk of mortality may be increased when corticosteroids or immunosuppressants were used in patients with malignancies.

This study has several limitations. First, we did not have access to individual clinical records, as we analyzed a database of COVID-19-related claims, and we are unable to determine why the patients received corticosteroids or immunosuppressants. In addition, we could not determine whether disease activity was considered acute or chronic, or whether the malignancies involved stable disease, disease progression, or distant metastasis. Second, we only included a small number of patients with hematological malignancies, HIV/AIDS, and organ transplantation, which precluded related subgroup analyses. Moreover, immunocompromised status could be mainly influenced by solid tumors or the use of corticosteroids. Nevertheless, we evaluated a large sample of patients from the national health insurance database by performing IPTW minimized the loss of population to consider generalizability and accuracy, and the PS-based IPTW analysis likely helped improve the reliability of the results.

## Conclusions

Immunocompromised Korean patients with COVID-19 had higher risks of severe illness and mortality, relative to non-immunocompromised patients with COVID-19. In particular, poor outcomes were associated with previous corticosteroid use. Therefore, caution is likely warranted when managing patients with COVID-19 who have a history that includes immunosuppressive conditions and/or treatments.

## Supporting information

S1 Fig Study flowchart. Immunocompromised status was identified based on a diagnosis of malignancy, a diagnosis of HIV/AIDS, organ transplantation within 3 years, prescribed corticosteroids or oral immunosuppressants for ≥30 days during the last year, and prescribed non-oral immunosuppressants at least once during the last year. COVID-19: Coronavirus disease 2019; HIV: Human immunodeficiency virus; AIDS: Acquired immune deficiency syndrome.
(DOCX)

S1 Table Specific parameters for identifying immunocompromised status. (DOCX)

S2 Table Outcomes in the subgroups of immunocompromised COVID-19 patients. (DOCX)

S3 Table Adjusted risks of outcomes among the COVID-19 patients with solid tumors. (DOCX)
