# Supplementary material for: COVID-19-related outcomes in immunocompromised patients: A nationwide study in Korea
Source: PLoS One. 2021 Oct 1;16(10):e0257641. doi: 10.1371/journal.pone.0257641 (PMC8486114; doi:10.1371/journal.pone.0257641)
Supplement: S1 Fig — Immunocompromised status was identified based on a diagnosis of malignancy, a diagnosis of HIV/AIDS, organ transplantation within 3 years, prescribed corticosteroids or oral immunosuppressants for ≥30 days during the last year, and prescribed non-oral immunosuppressants at least once during the last year. COVID-19: Coronavirus disease 2019; HIV: Human immunodeficiency virus; AIDS: Acquired immune deficiency syndrome. (DOCX) [file pone.0257641.s001.docx]

S1 Fig. Study flowchart. Immunocompromised status was identified based on a diagnosis of malignancy, a diagnosis of HIV/AIDS, organ transplantation within 3 years, prescribed corticosteroids or oral immunosuppressants for ≥30 days during the last year and prescribed non-oral immunosuppressants at least once during the last year.

COVID-19: coronavirus disease 2019; HIV: human immunodeficiency virus; AIDS: acquired immune deficiency syndrome.

Non-immunocompromised COVID-19
(n=5,564, 86.5%)

Immunocompromised COVID-19
(n=871, 13.5%)

Confirmed COVID-19, age of ≥18 years (n=6,435)

Immunocompromised COVID-19

Non-immunocompromised COVID-19

COVID-19 test (n=234,427)

Propensity score-based inverse probability of treatment weighting analysis
