# Supplementary material for: COVID-19-related outcomes in immunocompromised patients: A nationwide study in Korea
Source: PLoS One. 2021 Oct 1;16(10):e0257641. doi: 10.1371/journal.pone.0257641 (PMC8486114; doi:10.1371/journal.pone.0257641)
Supplement: S1 Table — (DOCX) [file pone.0257641.s002.docx]

**S1 Table. Specific parameters for identifying immunocompromised status**

| **Variable** | **Descriptions** |
| --- | --- |
| Malignancy | KCD 7 codes: C00-C97 |
| HIV or AIDS | KCD 7 codes: B20.x–B22.x, B24.x |
| Corticosteroids | Deflazacort, dexamethasone, betamethasone, hydrocortisone, methylprednisolone, prednisolone, triamcinolone, fludrocortisone, prednisone |
| Immunosuppressant | Abatacept, adalimumab, aldesleukin, alemtuzumab, anakinra, arsenic trioxide, asparaginase, azacitidine, azathioprine, basiliximab, bendamustine, bevacizumab, bleomycin, bortezomib, brentuximab, busulfan, cabazitaxel, capecitabine, carboplatin, carfilzomib, certolizumab pegol, cetuximab, cisplatin, cladribine, clofarabine, cyclophosphamide, cyclosporine, cytarabine, dacarbazine, dasatinib, decitabine, denosumab, dexrazoxane, docetaxel, doxorubicin, eculizumab, epirubicin, eribulin mesylate, erlotinib, estramustine phosphate sodium, etanercept, etoposide, everolimus, fingolimod, fludarabine phosphate, fluorouracil, gefitinib, gemcitabine, glatiramer acetate, golimumab, idarubicin, ifosfamide, imatinib, infliximab, interferon alfa-2a, interferon beta-1a, irinotecan, lapatinib, leflunomide, lenalidomide, melphalan, mercaptopurine, mesna, methotrexate, mitomycin, mitoxantrone, mycophenolate, natalizumab, nilotinib, oxaliplatin, palivizumab, pazopanib, peginterferon, pemetrexed, pertuzumab, pimecrolimus, rituximab, secukinumab, sirolimus, sorafenib, sunitinib, tacrolimus, temozolomide, temsirolimus, thalidomide, thiotepa, tocilizumab, topotecan, trastuzumab, tretinoin, ustekinumab, vinblastine, vincristine, vinorelbine |
| Organ transplantation | EDI codes: R3272,R3275,R3280, Q8030-Q8037, Q8040-Q8052, Q8061, Q8062, Q8070, Q8080, Q8091, Q8092, Q8101-Q8103, Q8111, Q8112, Q8121-Q8123, Q8140-Q8150, X5020, X5051, X5061-X5064, X5111-X5115, X5120, X5131-X5136, X6001-,X6008, X7000, X7001 |

KCD-7: Korean Classification of Disease, 7th edition; HIRA: health insurance review agency; HIV: human immunodeficiency virus; AIDS: acquired immune deficiency syndrome, EDI: Electronic Data Interchange.

* Immunocompromised status was identified based on a diagnosis of malignancy, a diagnosis of HIV/AIDS, organ transplantation within 3 years, prescribed corticosteroids or oral immunosuppressants for ≥30 days during the last year, and prescribed non-oral immunosuppressants at least once during the last year.
