# Supplementary material for: COVID-19-related outcomes in immunocompromised patients: A nationwide study in Korea
Source: PLoS One. 2021 Oct 1;16(10):e0257641. doi: 10.1371/journal.pone.0257641 (PMC8486114; doi:10.1371/journal.pone.0257641)
Supplement: S2 Table — (DOCX) [file pone.0257641.s003.docx]

**S2 Table. Outcomes in the subgroups of immunocompromised COVID-19 patients**

| **Outcomes** | **Malignancy**  **(n=515)** | **HIV/AIDS**  **(n=7)** | **Organ transplantation**  **(n=15)** | **Use of corticosteroids**  **(n=341)** | **Use of**  **immunosuppressants**  **(n=128)** | **≥2 causes**  **(n=117)** |
| --- | --- | --- | --- | --- | --- | --- |
| In-hospital mortality | 47 (9.1) | 1 (14.3) | 8 (53.3) | 36 (10.6) | 14 (11.7) | 19 (16.2) |
| Conventional oxygen therapy | 139 (27.0) | 2 (28.6) | 12 (80.0) | 87 (25.5) | 35 (29.2) | 42 (35.9) |
| High flow nasal cannula | 38 (7.4) | 0 (0.0) | 10 (66.7) | 22 (6.5) | 12 (10.0) | 15 (12.8) |
| Mechanical ventilation | 24 (4.7) | 0 (0.0) | 13 (86.7) | 13 (3.8) | 6 (5.0) | 9 (7.7) |
| ECMO | 2 (0.4) | 0 (0.0) | 4 (26.7) | 1 (0.3) | 0 (0.0) | 0 (0.0) |
| Vasopressor use | 39 (7.6) | 0 (0.0) | 2 (13.3) | 21 (6.2) | 16 (13.3) | 16 (13.7) |
| Renal replacement therapy | 5 (1.0) | 0 (0.0) | 5 (33.3) | 4 (1.2) | 2 (1.7) | 3 (2.6) |
| Acute heart failure | 57 (11.1) | 0 (0.0) | 4 (26.7) | 27 (7.9) | 10 (8.3) | 12 (10.3) |

Data are shown as number (%).

COVID-19: coronavirus disease 2019; IPTW: inverse probability of treatment weighting; HIV: human immunodeficiency virus; AIDS: acquired immune deficiency syndrome; ECMO: extracorporeal membrane oxygenation.
