# Supplementary material for: COVID-19-related outcomes in immunocompromised patients: A nationwide study in Korea
Source: PLoS One. 2021 Oct 1;16(10):e0257641. doi: 10.1371/journal.pone.0257641 (PMC8486114; doi:10.1371/journal.pone.0257641)
Supplement: S3 Table — (DOCX) [file pone.0257641.s004.docx]

**S3 Table. Adjusted risks of outcomes among the COVID-19 patients with solid tumors**

| **Outcomes** | **Solid tumor before PS-based IPTW (n=503)** | **Solid tumor after PS-based IPTW (n=3,269)** | **Adjusted OR ***  **(95% CI)** | ***p*-value** |
| --- | --- | --- | --- | --- |
| In-hospital mortality | 46 (9.2) | 181 (5.5) | 1.26 (0.94–1.69) | 0.120 |
| Conventional oxygen therapy | 137 (27.2) | 608 (18.6) | 1.03 (0.89–1.20) | 0.653 |
| High flow nasal cannula | 37 (7.4) | 154 (4.7) | 0.84 (0.62–1.13) | 0.253 |
| Mechanical ventilation | 25 (4.8) | 62 (1.9) | 0.67 (0.45–1.00) | 0.051 |
| ECMO | 2 (0.4) | 0 (0.0) | NA |  |
| Vasopressor use | 39 (7.8) | 141 (4.3) | 1.26 (0.93–1.71) | 0.140 |
| Renal replacement therapy | 5 (0.1) | 11 (0.4) | 0.35 (0.12–1.03) | 0.058 |
| Acute heart failure | 54 (10.7) | 319 (9.8) | 1.41 (1.17–1.71) | 0.003 |

Data are shown as number (%).

COVID-19: coronavirus disease 2019; IPTW: inverse probability of treatment weighting; OR: odds ratio; CI: confidence interval; ECMO: extracorporeal membrane oxygenation; NA: not applicable.

* Adjusted for Charlson comorbidity index, age, and region.
